# A new route to obtain fluorescence X-ray absorption spectra of compounds and to remove the self-absorption induced nonlinearity in the spectra

**DOI:** 10.1107/S1600577522000029

**Published:** 2022-02-08

**Authors:** Qing Ma, Stephanie L. Moffitt, Denis T. Keane

**Affiliations:** a Northwestern Synchrotron Research Center at the Advanced Photon Source, Argonne, IL 60439, USA; b Materials Science and Engineering Department, Northwestern University, Evanston, IL 60208, USA

**Keywords:** X-ray absorption spectroscopy, self-absorption effect, amorphous transparent conducting oxides

## Abstract

A new route to obtain fluorescence X-ray absorption spectra of compounds and to remove the self-absorption induced nonlinearity in the spectra is described. The fluorescent intensity *I*_f_ is linearly proportional to the absorption coefficient μ. For studies of surface structures around an element (κ) the fluorescence detection is often the mode of choice. However, the measurement may suffer from a self-absorption (SA) effect which nonlinearly distorts the spectra. The effect is severe when κ is concentrated or the measurements are carried out in certain geometries. Here, the correlations among emission events in compounds are examined following resonance X-ray core-electron excitation within κ. Under conditions leading to SA, *I*_f_ emitted from κ apparently has a conjugated relationship with the fluorescent intensities simultaneously emitted from other elements (ξ). Normalizing the former (κ) by the latter (ξ) will largely remove SA effects and reduce this nonlinear problem to a tractable linear problem. This does result in a moderate reduction of the spectral amplitude due to the so-called secondary emission from ξ excited by the emission from κ. Nonetheless, the resulting spectra will allow one to accurately determine bond distances and disorder and, in some respects, can be superior to spectra obtained *via* the absorption channel. For μ_ξ_ < μ_κ_ and grazing incidence geometry, the amplitude reduction can be small and simple normalization is sufficient to restore the spectral integrity with remarkable accuracy. This has been instrumental in unravelling the surface and subsurface structures around cations in amorphous Ga–In–O and Zn–Sn–O films which are otherwise inaccessible due to severe SA effects. This method has also been applied to several samples with μ_ξ_ ≃ μ_κ_ to examine its applicability. For these samples, the amplitude reduction is 12 ± 4% versus their standards for the data measured with the classical 45°/45° geometry. This experimental method is easy to implement. Since *I*_f_ from κ and ξ are measured by the same detector system, it is also superior to other methods in removing systematic errors such as detector system nonlinearity, electronic noise, and some beam instabilities, and in removing spectral imperfections due to, for example, SA effects, diffraction effects and sample inhomogeneity. The distortions resulting from the latter can be severe in the spectra measured in transmission mode.

## Abstract 2

This work describes an experimental method in tackling the self-absorption effect encountered in fluorescence X-ray absorption spectroscopy and its application to surface and subsurface.

## 1. Introduction

There has been an increasing interest in amorphous transparent conducting oxides (a-TCO) for applications of transparent electrodes in flat panel displays, solar cells, and light emitting diodes since they have electrical and optical properties comparable with their crystalline counterparts (Ginley *et al.*, 2011 ▸). Our primary interest is to study the surface and subsurface atomic and electronic structures of a-TCO films, rarely addressed, using fluorescence X-ray absorption spectroscopy (XAS) techniques (Moffitt *et al.*, 2018 ▸). Modern core-shell XAS techniques are powerful tools to peer into the electronic and atomic structures around a specific element in materials systems. These techniques, namely X-ray absorption near-edge structure (XANES) and extended X-ray absorption fine structure (EXAFS), are now widely used due largely to easy access to synchrotron radiation facilities worldwide, contributing greatly to our understanding of materials chemistry and physics in atomistic detail. Among various modes of measurement, *e.g.* transmission, fluorescence, and electron yield, the fluorescence mode is arguably the most versatile. However, *extreme* caution should be taken to minimize so-called self-absorption (SA) effects (Goulon *et al.*, 1982 ▸) that result in spectral distortion. For a bulk material, a thin layer of powder may be used for its measurement to avoid the SA effect. The large incidence and grazing emission geometry may be used to measure *flat* samples (Becker *et al.*, 1983 ▸). The ultra-grazing incidence geometry may be used to study the *smooth* surfaces of thin films (Moffitt *et al.*, 2018 ▸). The goal of the present work is to introduce a new method to remove the SA-induced spectral nonlinearity experimentally. It will be applied first to the systems of our interest and then be generalized for other systems.

We have used the ultra-grazing incidence angle technique to study the smooth surfaces of a-TCO films (Moffitt *et al.*, 2016 ▸, 2018 ▸). It was found that the SA effect was absent when the X-ray incidence angle α ≤ 2/3*θ*_c_, where *θ*_c_ is the critical angle. But it is severe when α ≃ θ_c_, which prohibits access to the subsurface. For samples with rougher surfaces and large concentrations of the element of interest, it is impossible to measure surface structure, let alone subsurface structure, due to the SA effect which causes nonlinear attenuation of the XAS spectra. It may be corrected to some extent using the methods proposed in the past (Pfalzer *et al.*, 1999 ▸; Tröger *et al.*, 1992 ▸; Eisebitt *et al.*, 1993 ▸; Booth & Bridges, 2005 ▸; Trevorah *et al.*, 2019 ▸; Haskel, 1999 ▸). A good summary and comments about these methods are given by Trevorah *et al.* (2019 ▸) who also include extensive citations of further literature. All these methods require detailed information about instruments and samples or are limited to certain geometries. The major ones are implemented in various software packages, such as the popular *Athena* package (Ravel & Newville, 2005 ▸). The methods were applied to the data presented here with little success. Recently, Achkar *et al.* (2011 ▸) reported an inverse partial fluorescence yield (IPFY) method to experimentally remove SA effects. In this method, the XAS data around the element of interest (κ) is obtained by inversion of a simultaneous emission from another element (ξ). Unfortunately, it is flawed, which becomes evident when applied to our surface XAS data presented here or to samples of less than two optical thicknesses. This motivated us to search for a new route to obtain fluorescence spectra and to remove SA-induced nonlinearity in the XAS spectra. In the method described here, the emission from ξ is used to normalize the emission from κ. As will be shown, under grazing incidence conditions simple normalization is sufficient to remove SA effects and to satisfactorily restore the spectral integrity. This is instrumental in unravelling surface and subsurface structures of amorphous Ga–In–O films. We will briefly describe the SA effect, examine the correlations among various emissions following X-ray excitation, and present an analytical description of the method. We will then apply it to other systems and discuss its applicability for structural studies of material systems in general.

X-ray photons propagating through matter are subject to absorption. After considering the absorption effect on the intensities of both the incident X-ray beam *I*_0_ at energy *E* and of the X-ray induced fluorescence of energy *E*_f_, the exiting fluorescence intensity *I*_f_ is written as

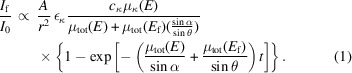




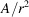
 is the detecting solid angle (Ω); ε_κ_ is the probability of the fluorescence decay; 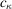 and 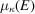 are the concentration and absorption coefficient; μ_tot_(*E*) and μ_tot_(*E*_f_) are the total μ (= 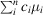 ) at *E* and *E*_f_; θ is the detecting angle; *t* is the sample thickness. In the thin sample limit, including the cases where α < θ_c_, equation (1) reduces to 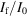 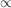 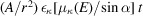 . Thus, *I*_f_ is linearly proportional to 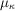 , and contains no SA effects.

For thick samples and concentrated elements of interest the exponent term ≃ 0 and equation (1) reduces to

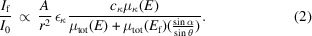

The spectra will be attenuated through cancellation due to the large 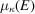 contribution to μ_tot_(*E*) or suffer from SA effects. For α near 90°, μ_tot_(*E*) is negligible in equation (2) so the SA is at a minimum.

## 2. Experimental section

X-ray absorption measurements were carried out at the bending magnet beamline 5-BM-D at the Advanced Photon Source (Argonne, IL, USA). A Si(111) double-crystal monochromator was used for energy selection (Δ*E*/*E* = 1.4 × 10^−4^). The incident X-ray intensity was 60% detuned for harmonic rejection and monitored by an ion chamber filled with gases absorbing 10% of the incident X-rays. Fluorescence measurements were carried out using two four-element Si-drift solid-state detectors (Hitachi, USA) equipped with the xMAP digital pulse processors (XIA LLC). The dead-time effect is carefully avoided by limiting the count rates well within the dead-time correctable range by adjusting the detector-to-sample distance and changing the incident beam size. Transmission measurements were carried out using ionization chambers (FMB Oxford). The ion chambers before and after the sample were filled with 600 He/100 N_2_ (Torr) and 1150 He/250 N_2_ (Torr), respectively, to ensure similar response characteristics. For amorphous film samples, the XAS spectra were collected in fluorescence mode in the grazing incidence geometry as a function of α. The samples of size 10 mm × 20 mm were placed on a two-circle Huber rotation stage with surface normal upward and the long side along the X-ray beam direction. The detectors were placed 90° to the beam direction and above the film surface with θ ≃ 20° on average. The beam size was set to 0.05 mm × 8 mm and 0.02 mm × 8 mm (vertical × horizontal) for the low- and high-edge energies, respectively. A second set of slits was placed behind the sample for alignment using an ion chamber which is also used to measure the X-ray reflectivity (XRR). For bulk samples, the powder-on-tape samples were held vertically with the X-ray incidence angle at 45° to the samples and were measured simultaneously in transmission and fluorescence modes. Crystalline wafer samples were mounted on a spinner and were spun to minimize diffraction effects during data collection. A rotation stage (Newport) was used to vary the α angles.

Like the IPFY method, both the resonance emission from κ and a simultaneous non-resonance emission (NRE) from ξ are measured, *e.g.* for the In *K*-edge XAS, the In and Ga *K_α_* emissions are measured simultaneously while the incident X-ray energy scans across the In *K*-edge.

The amorphous Ga(10 at.%)–In–O and Zn(30 at.%)–Sn–O films (*t* ≃ 250 and 400 nm) used in this study were previously prepared by pulsed laser deposition and the details can be found elsewhere (Buchholz *et al.*, 2014 ▸; Moffitt *et al.*, 2017 ▸). Fig. S1 of the supporting information shows the film surfaces characterized by X-ray reflectivity and fluorescence. The differences between the films can be attributed to surface roughness. A characteristic of these film systems is the large contrast in elemental concentration and absorption cross-sections. *e.g.* 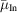 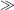 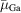 , where 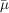 = ε*c*μ, for the In *K*-edge XAS. For the systems with 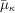 ≃ 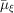 we use a GaAs wafer of 0.5 mm thickness, GaAs powder from the wafer, and a commercial CuSe powder for method validation and generalization. The advantage of using a crystalline wafer is its warranted uniformity. However, diffraction effects could be a concern and therefore we mitigated these by using a sample spinner. There are still faint tell-tale signs of some diffraction peaks that may exist in high-energy regions, but they are too small to be a concern. To prepare the GaAs powder, a piece of wafer was crushed and ground into fine powder. The powder that naturally fell through a 50 µm mesh was collected and prepared on a long Scotch tape (3M, Inc). The quality of the tape sample was characterized by measuring the variation of the transmitted X-ray intensities (Fig. S2). The prepared powder-on-tape GaAs sample is clearly suitable to be used as standard. For CuSe, it was not possible to prepare quality tape samples (Fig. S3), which however is useful to illustrate common problems with sample inhomogeneity and the power of the method described here in handling them.

## 3. Results and discussions

**Figure 1:**
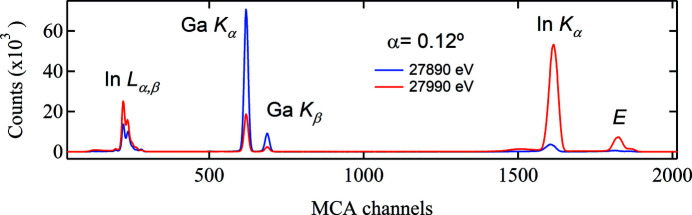
The MCA spectra measured below and above the In *K*-edge (27940 eV). The quantum efficiency of the detector is not considered (which is Ga *K_α_*:In *K_α_* ≃ 10:1).

Fig. 1 ▸ shows the multichannel analyzer (MCA) spectra recorded at two energies, *i.e.* 50 eV below and above the In *K* absorption edge (27940 eV), on the Ga–In–O film.

The X-ray emission process is complex. For the Ga–In–O film excited by X-rays with energy above the In *K*-edge, at a minimum the following photoexcitation and emission processes proceed,

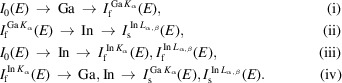

The subscript ‘s’ denotes *I*_f_-induced emissions or secondary emissions. For an X-ray energy near but below the In *K*-edge (Fig. 1 ▸), the processes (i) and (ii) dominate fluorescent events; 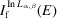 in (iii) is negligibly small due to the cross-section effect. Above the In *K*-edge, processes (iii) and (iv) produce modulated emissions due to the XAS process. Therefore, 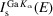 and 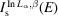 carry innately the features of 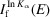 . The Ga *K_α_* emission is thus composed of 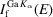 and 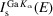 and the In *L*_α,β_ emission of 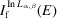 and 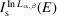 . However, the secondary Ga *K*_α_ emission is an interatomic event, and its magnitude depends on concentration and cross-sections. The secondary In *L*_α,β_ emission is mainly an intra-atomic event, as will be confirmed later. Their intensities respond differently to the energy change around the In *K*-edge (Fig. 1 ▸). It is estimated that 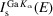 is a fraction of 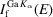 for the Ga–In–O film in which Ga is a minor component, and its quantum yield due to 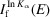 is <<1% [ 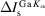 / 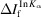 , the edge height ratio]. Thus, 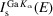 is a rare event in this case. For the samples in which the ξ content is high, the secondary emission can be non-negligibly significant as will be shown later for a GaAs wafer for which 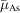 ≃ 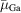 .

### 3.1. Experimental method to remove the SA-induced spectral nonlinearity

**Figure 2:**
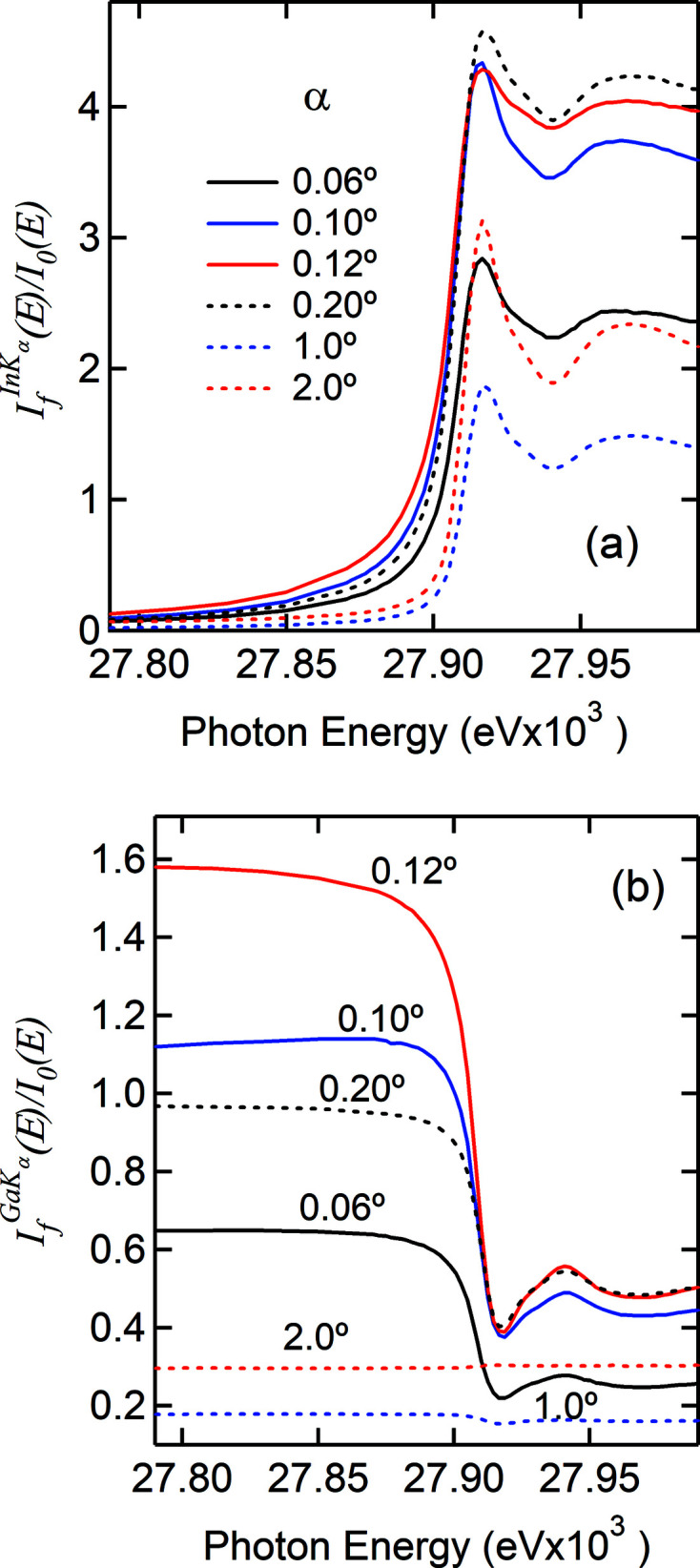
Raw In *K_α_* spectra (*a*) and raw Ga *K_α_* spectra (*b*) as a function of the X-ray incidence angle α, measured simultaneously while the X-ray energy scans across the In *K*-edge.

Given the large In concentration and the rough surface of the Ga–In–O film, the SA effect makes it impossible to properly carry out the XAS studies of the local structures around surface or subsurface In sites. Figs. 2 ▸(*a*)–2(*b*) show as a function of α the raw spectra of the In *K_α_* and Ga *K_α_* emissions measured on the Ga–In–O film. In addition to the attenuation of the XAS oscillations, the elevation of the pre-edge region is also a fingerprint of SA effects in XAS spectra. As is seen in Fig. 2 ▸(*a*), the SA effect becomes severe from low angles to α ≃ θ_c_ (0.12°) and thereafter decreases as α increases and the measurement geometry approaches the thin film limit. It vanishes when the Ga *K_α_* emission shows a positive step (*e.g.* α = 2°). For both Ga–In–O and Zn–Sn–O films the In and Sn *K*-edge spectra measured at α > 1° is free of SA effects. Thus, the NRE can be used as a reliable, on-the-fly check on the SA effect. Since 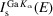 is a rare event here, it shall not suffer from SA effects. For high concentration, we show later that the secondary emission can even be SA attenuated. It is therefore important to evaluate it, since it will impact the accuracy of the method introduced here. Unfortunately, it is inseparable from the SA effect, which complicates its accurate measurements and direct evaluation in most cases.

**Figure 3:**
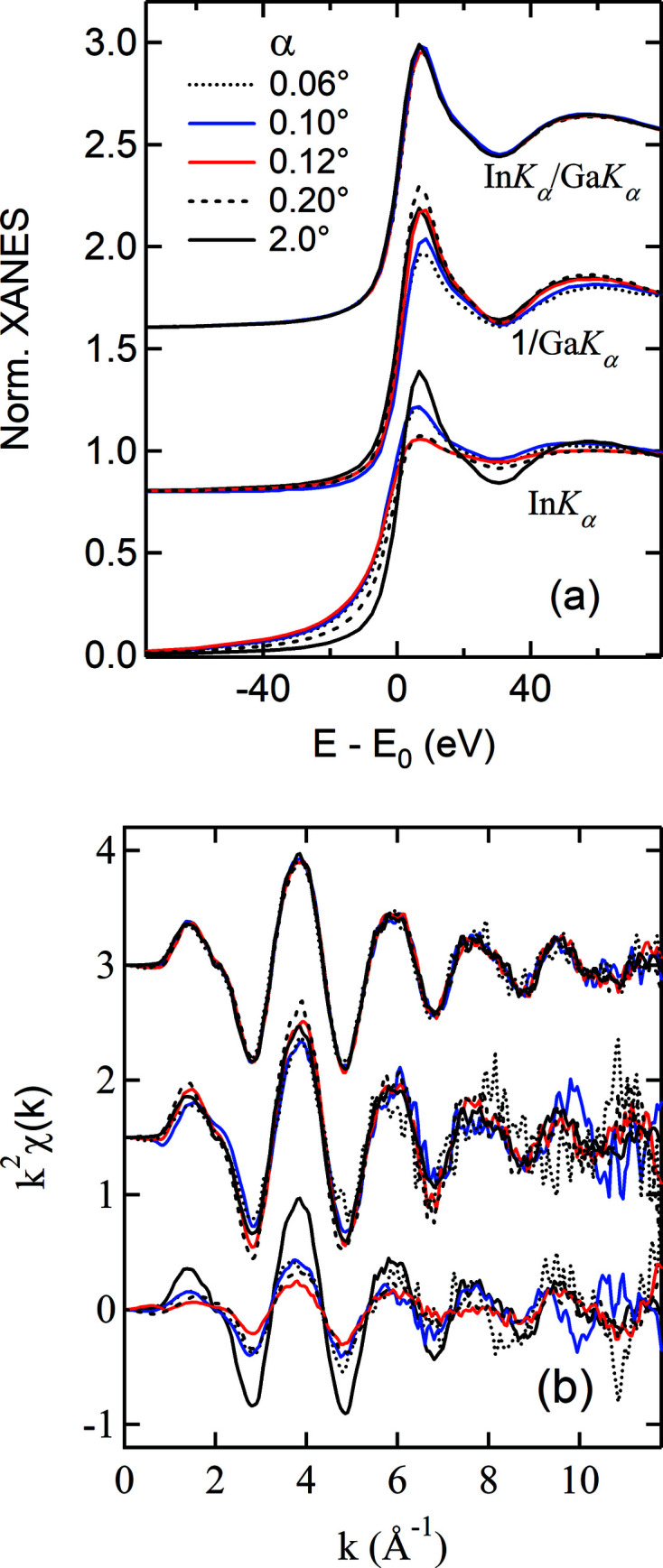
The In *K*-edge XANES (*a*) and EXAFS (*b*) obtained *via* three methods: In *K_α_*, 1/Ga *K_α_*, and In *K_α_*/Ga *K_α_*. See Fig. S4 for the Fourier transforms of EXAFS.

At first glance, the modulations seen on the *E*-dependence of the NRE [Fig. 2 ▸(*b*)] follow closely the XAS modulations *nearly* as a mirror image when the SA effect is present, but in phase when it is absent. From Fig. 2 ▸ it is clear that the drop in 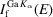 at the onset of the In *K*-edge correlates with the severity of the SA effect and is due to a large increase in 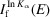 which itself is SA suppressed. In other words, both 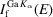 and 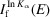 are attenuated under SA conditions. We are therefore motivated to normalize 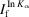 by 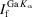 . Fig. 3 ▸ shows such normalized results, compared with the ones obtained conventionally from 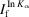 and from 1/ 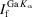 (the IPFY method), respectively. It is quite astonishing that the nonlinearity due to SA effects has vanished and that the spectra compare remarkably well with the one measured at α = 2° that shows little sign of SA effects. These results manifest in an unprecedented way that it is indeed possible to experimentally remove the SA effect and illustrate the intrinsic correlations among emission events following core-electron excitation. It may be readily concluded that the local structure around In from surface to subsurface is similar to that in the film bulk (Moffitt *et al.*, 2017 ▸). The results for the Zn (∼30%)–Sn–O film are equally compelling and presented in detail in Fig. S6.

On the other hand, the results from the IPFY method appear to be sporadic, which is not surprising since 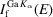 contains SA-induced distortion as much as 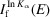 . The results in Fig. 3 ▸ indicate that the oscillations in 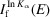 and 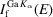 are likely a conjugated pair. In addition to the discrepancies in magnitudes, such obtained XANES all show narrow edges, which would imply a longer lifetime of the In 1*s* core-hole and is unsettling. The fundamental issue is that the IPFY method is susceptible to a thickness effect, as illustrated later. Moreover, 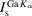 is not considered in the IPFY method which may result in non-negligible errors in the magnitude when the NRE is strong. We would also like to point out a problematic statement given by Achkar *et al.* (2011 ▸) about using the lower-energy threshold emissions to obtain the emission data of the higher-energy threshold from the same element. As pointed out above, In *L_α,β_* responds differently from Ga *K_α_* to the energy change across the In *K*-edge (Fig. 1 ▸) and varies linearly with 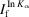 . It produces a replica of the In *K*-edge XAS (Fig. S5). This is because above the In *K*-edge the increase in the In *L* emission is due to the intra-atomic electronic processes, *i.e.* the 2*p* hole left behind following the 2*p* → 1*s* de-excitation is filled by none other than 3*d* electrons of the same atom. It thus oscillates in step with the In *K_α_* emission. Therefore, the In *L* emission cannot be used for the SA removal in the In *K*-edge spectra.

As seen in Fig. 3 ▸(*b*), the oscillations extracted from 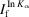 and 1/ 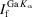 show some irregularies in the high-*k* region, in particular, for the data measured at small angles (α = 0.06° and 0.10°). They have a phase relationship of ∼π; opposite to the SA effects. We attribute these irregularities to a combination of surface roughness and potential beam instability. They have disappeared in the normalized data. Therefore, the normalizion method has an added benefit, *i.e.* being effective in removing non-EXAFS-related features. Naturally, the SA-induced spectral distortions are non-EXAFS-related features.

We now examine the underlying mechanism of this normalization method and its limit. Equation (2) can be written both for the In and Ga *K_α_* emissions, respectively,

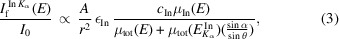




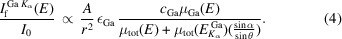

The results in Fig. 3 ▸ may thus be described by the ratio of equation (3) to equation (4),

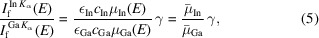

where

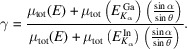

In the grazing incidence geometry, ( 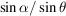 ) is small and thus γ ≃ 1. Therefore, μ_tot_(*E*) is removed which is the source of SA effects. We now have

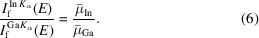

Since μ_Ga_(*E*) is quasi *constant* around the In *K*-edge, equation (6) is linearly proportional to μ_In_(*E*). Table 1 ▸ shows the ratios of the second-to-first terms in the denominators of equations (3) and (4), derived from the experimental conditions. Both are smaller than 2%, which thus validates equation (6) and the results shown in Fig. 3 ▸. The ratio can be further reduced using larger θ so that better accuracy is achieved. Despite high Zn content, equation (6) is also suited for the Zn–Sn–O film, as discussed in Section S6. Therefore, this method should represent a breakthrough since it allows access to the surface and subsurface structures around concentrated elements, which is otherwise inaccessible by the XAS technique due to SA effects.

We thus have shown that the normalization method is powerful for thin film systems that require grazing incidence geometry. Note that the X-ray incident intensity is no longer in equations (5) and (6). The data are therefore measured by the same detecting system. This is advantageous since: (1) electronic noise and spectral distortions due to factors such as sample inhomogeneity and diffraction effects can be reduced or removed and (2) systematic errors such as the dead-time effect associated with pulse-counting electronics and potential beam instabilities are cancelled. Noticeable improvements in data quality after the normalization [see Fig. 3 ▸(*b*)] are clearly due to such cancellations. Moreover, the solid-angle related term is also dropped, which is a required term for all the other self-absortion correction methods (Trevorah *et al.*, 2019 ▸; Brewe *et al.*, 1994 ▸). We will further substantiate these advantages with the measurements of bulk samples.

### 3.2. Method generalization

**Figure 4:**
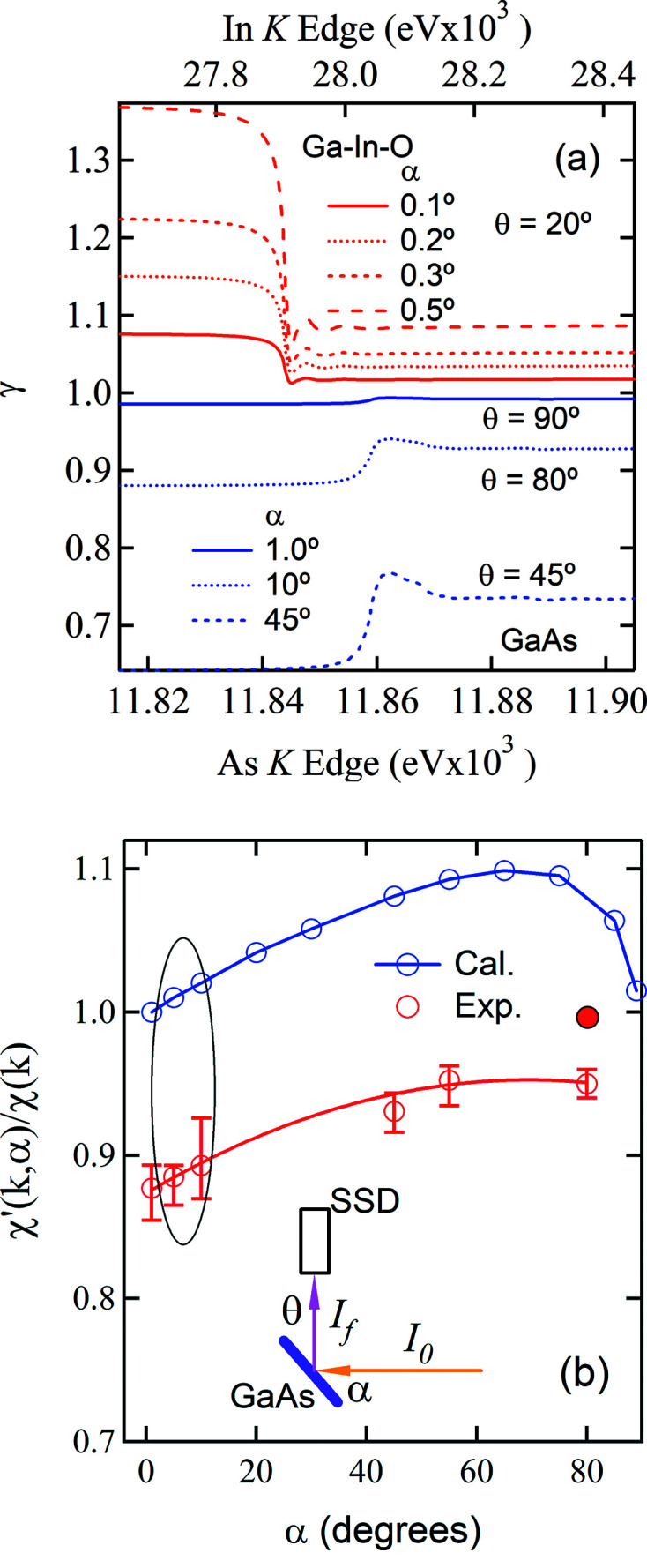
(*a*) The γ factor around the In and As *K*-edges as functions of α and θ (see details in Table S1 to S2). (*b*) The α-dependences of the γ effects on the EXAFS amplitudes (Calc.) and of As *K_α_*/Ga *K_α_* for the GaAs wafer (Exp.). The full red circle is the result from As *K_α_*. The oval trace indicates the region where the γ effect is small but the SA effect is large. Insert: experimental geometry.

From above, it is clear that the geometry can significantly influence the extent of the SA effects (Zschech *et al.*, 1992 ▸). It is the leading factor that limits applicability of many SA correction schemes (Booth & Bridges, 2005 ▸; Brewe *et al.*, 1994 ▸; Trevorah *et al.*, 2019 ▸). As part of generalizing the method, it is imperative to have a better understanding of the geometry-related γ factor. Fig. 4 ▸(*a*) shows the α-dependences of γ obtained using the SA-free spectra measured, respectively, at α = 5° on the Ga–In–O film and on powder GaAs in transmission mode. The details and the method to obtain μ_tot_(*E*) are presented in Tables S1–S3 in Section S7. Depending on *c* and μ, γ may show a negative or positive step. Fig. 4 ▸(*b*) is used to show the γ effects on the EXAFS amplitudes [equation (5)]. To do so without complications of 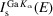 and SA effects in the measured 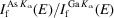 for the GaAs wafer, the spectrum μ(*E*) of the GaAs powder is used for this purpose. As shown in Fig. S7(*b*), *e.g.* for α = 10° and 45°, where the EXAFS amplitudes χ′(*k*, α) extracted from γ(α, *E*)μ(*E*) is compared with that of the powder χ(*k*), the γ effect on χ(*k*) is clearly seen. Scaling χ(*k*) to χ′(*k*, α) yields the results (blue open circles) presented in Fig. 4 ▸(*b*). The γ effect rises as α increases. As α and θ approach the α = 90° and θ = 0° geometry it vanishes, at which equation (5) will produce the data free of SA effects, just like that produced from equation (3) alone at this condition, *i.e.* for the grazing emergence technique to operate (Becker *et al.*, 1983 ▸). The α-dependence of the measured data is apparently governed primarily by the γ effect, which coincides with the geometry-related behaviour of the SA effect in the angular region near α = 90° described by equation (3). At the region near α = 0° it coincides with the thin-limit conditions described in the *Introduction* [Sec sec1]. For α = 0.1–0.5° and θ = 20°, γ results in a 0.9–2.0% reduction of the EXAFS amplitude for the Ga–In–O film, compared with an increase of ≤2% for α = 1.0–10° and θ = 89–80° and ∼8% for α = θ = 45° for GaAs [Fig. S7(*b*)].

**Figure 5:**
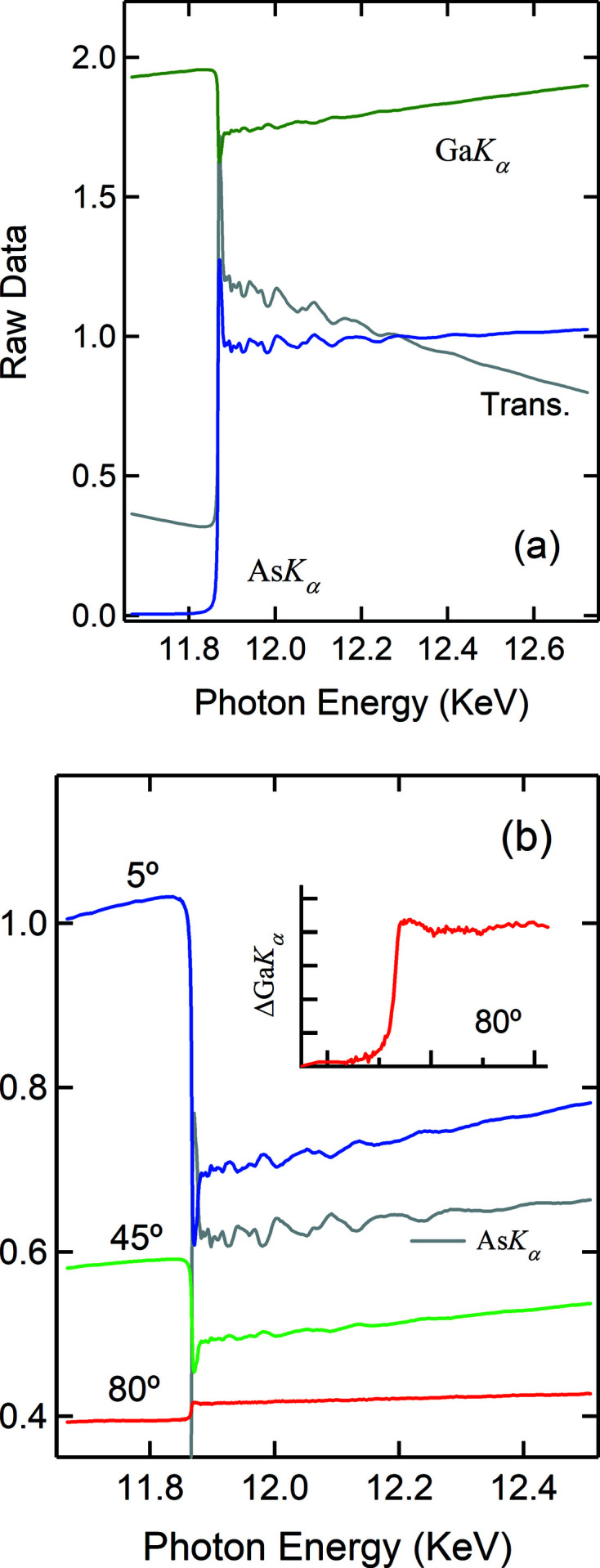
(*a*) Raw data measured on the GaAs powder, simultaneously, through the As *K_α_*, Ga *K_α_* and absorption channels at the As *K*-edge. (*b*) Raw Ga *K_α_* data measured on the GaAs wafer for various incidence angles. The As *K_α_* data at α = 5° is given to show its correlation with Ga *K_α_*. The insert shows the details of the secondary emission edge step induced by the As *K_α_* emisson.

Interestingly, the EXAFS amplitude from 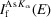 / 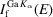 should approach that of the standard at α = 45° due to the γ effect alone. But this is not what we observe experimentally, which is likely because equations (5) and (6) did not consider the 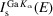 contribution. The data collected at α = 80° provide direct evidence for this conjecture. As will be shown in Fig. 5 ▸(*b*), this contribution is measured (the positive step for Ga *K_α_*) at α = 80° for the GaAs wafer where SA effects are negligibly small, and the As *K_α_* data are very close to the standard [full red circles in Fig. 4 ▸(*b*)]. Normalization of the As *K_α_* data by the Ga *K_α_* data results in an amplitude reduction of ∼5% at α = 80° [red open circles in Fig. 4 ▸(*b*)]. As will be demonstrated, the reduction is linear, or quasi-linear due to weak secondary emission contributions. For some applications where one is interested in bond distances and structural disorder (Sayers *et al.*, 1974 ▸), equation (5) should produce results with accuracies, in some practical respects, better than other methods.

Based on the above, the relation between the true EXAFS 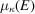 and the EXAFS from 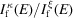 may therefore be represented by the following equation,

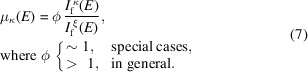

Note that the results from the IPFY method suffer from this amplitude reduction as well. This was not so obvious for thin films but will be clear for bulk samples.

To explore the applicability of the method in general, we discuss here the results measured on GaAs powder and wafer samples complemented by those on a powder sample of CuSe. From now on, we use the *X* *K*_α_ notation to replace the 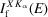 notation in most cases. Fig. 5 ▸ presents the As *K_α_* and Ga *K_α_* data measured on the GaAs powder (*a*) and wafer (*b*). The powder sample has an optical thickness of 0.75λ as indicated by the edge step of the transmission data. The absorption length is λ ≃ 11 µm above the As *K*-edge. As indicated by the dip in the Ga *K_α_* data and the smaller white line for the As *K_α_* data, the As *K_α_* data are SA attenuated. Nonetheless, the SA effect is moderate, when compared with the data measured on the GaAs wafer where the severity of the SA effect depends on geometry and increases quickly with the decrease of α. The positive step near the edge in the Ga *K_α_* data at α = 80° indicates that the SA effect is very weak or has vanished in the As *K_α_* data. The insert shows the details of the edge profile due to the secondary emission excited by As *K_α_*. Since the Ga concentration is high and the wafer can be considered *infinitely* thick, ΔGa *K_α_*/As *K_α_* is non-negligible and amounts to ∼5.5%. The white line is obviously attenuated. In Fig. 5 ▸(*b*) the As *K_α_* data are presented to better show its conjugated relationship with Ga *K_α_*. Note that to avoid the Ga *K_β_* emission, whose peak width overlaps partially with the As *K_α_* emission, only half of the emitted As *K_α_* intensity is measured by adjusting the detector integration energy range.

**Figure 6:**
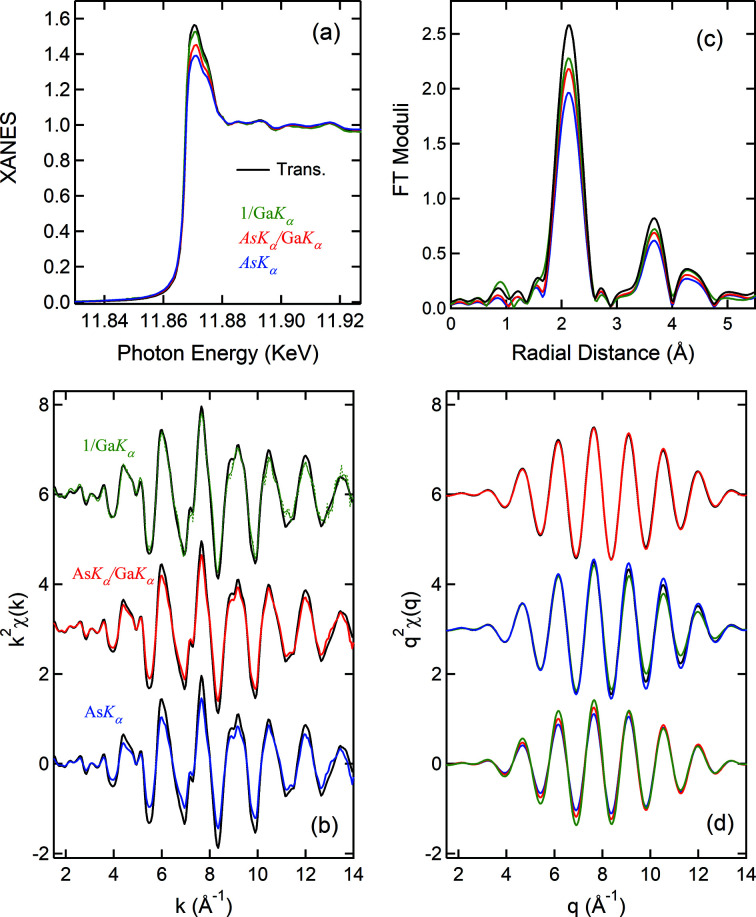
XAS spectra measured on GaAs powder, simultaneously, through the As *K_α_*, Ga *K_α_* and absorption channels at the As *K*-edge. (*a*) Normalized XANES. (*b*) Fluorescence EXAFS spectra compared with absorption EXAFS. The noise-filtered 1/Ga *K_α_* data are also shown (full green line). The FT filter cut-offs are *k* = 0–14 Å^−1^ and *R* = 0–10 Å. (*c*) The FT moduli of the EXAFS spectra (*k* = 3–12.4 Å^−1^, Hanning window). (*d*) The first-shell EXAFS obtained by the back FTs (*R* = 1.6–2.6 Å). Lower: As *K_α_* (blue), 1/Ga *K_α_* (green), and As *K_α_*/Ga *K_α_* (red). Middle: As *K_α_* and 1/Ga *K_α_* scaled to the standard in the low-*k* region. Upper: As *K_α_*/Ga *K_α_* data scaled to the standard by ×1.12.

Fig. 6 ▸ presents the processed data for the GaAs powder. For the white lines of the As *K*-edge, they are partially recovered by As *K_α_*/Ga *K_α_* but almost completely by 1/Ga *K_α_*, *i.e.* the IPFY method. For EXAFS data, the Ga *K_α_* data [dotted line, top of Fig. 6 ▸(*b*)] is considerably noisier despite being from a sum of 50 40-min scans, each of which is time weighted heavily in the high energy regions. The total counts in this region for Ga *K_α_* are ∼4.5 × 10^7^. Therefore this is a drawback to use the non-resonance emission alone (the IPFY method). The Fourier filtering [full green line, top of Fig. 6 ▸(*b*)] shows that in this case the long counting time was useful because the noise does not affect the overall EXAFS oscillations. As seen in Fig. 6 ▸(*b*), albeit weaker than that seen in Fig. 2 ▸(*b*), the As *K_α_* EXAFS show the typical characteristics of SA effects, *i.e.* the low-*k* oscillations are attenuated more than the high-*k* ones. On the other hand, 1/Ga *K_α_* shows an opposite trend due to their conjugate relationship. The Ga *K_α_* data are thus also distorted due to the SA effects.

Since the spectra are quite clean up to 12.5 Å^−1^ this range is used for the Fourier analyses [Figs. 6 ▸(*c*) and 6(*d*)]. Despite the similarity in the white-line height, the Fourier transform (FT) moduli [Fig. 6 ▸(*c*)] show that the peaks of the 1/Ga *K_α_* data are much smaller than those of the standard. In fact, it is very much comparable with the As *K_α_* data. Therefore, it is *a priori* with simple arithmetic that the SA-induced nonlinearity may be removed by our method. Using the back FT technique to isolate the dominating first-shell EXAFS better reveals the SA effect. The results are presented in three ways in Fig. 5 ▸(*d*): (bottom) the As *K_α_*, 1/Ga *K_α_*, and As *K_α_*/Ga *K_α_* data, (middle) the As *K_α_* and 1/Ga *K_α_* data scaled to the standard in the low-*k* region by ×1.40 and ×1.03, and (top) the As *K_α_*/Ga *K_α_* data scaled to the standard by ×1.12. The data (middle) show a clear dispersion with the wave number, which is obscured by the high-shell EXAFS which suffer less from the SA effects. It is clear that the As *K_α_*/Ga *K_α_* data contain essentially no distortions.

**Figure 7:**
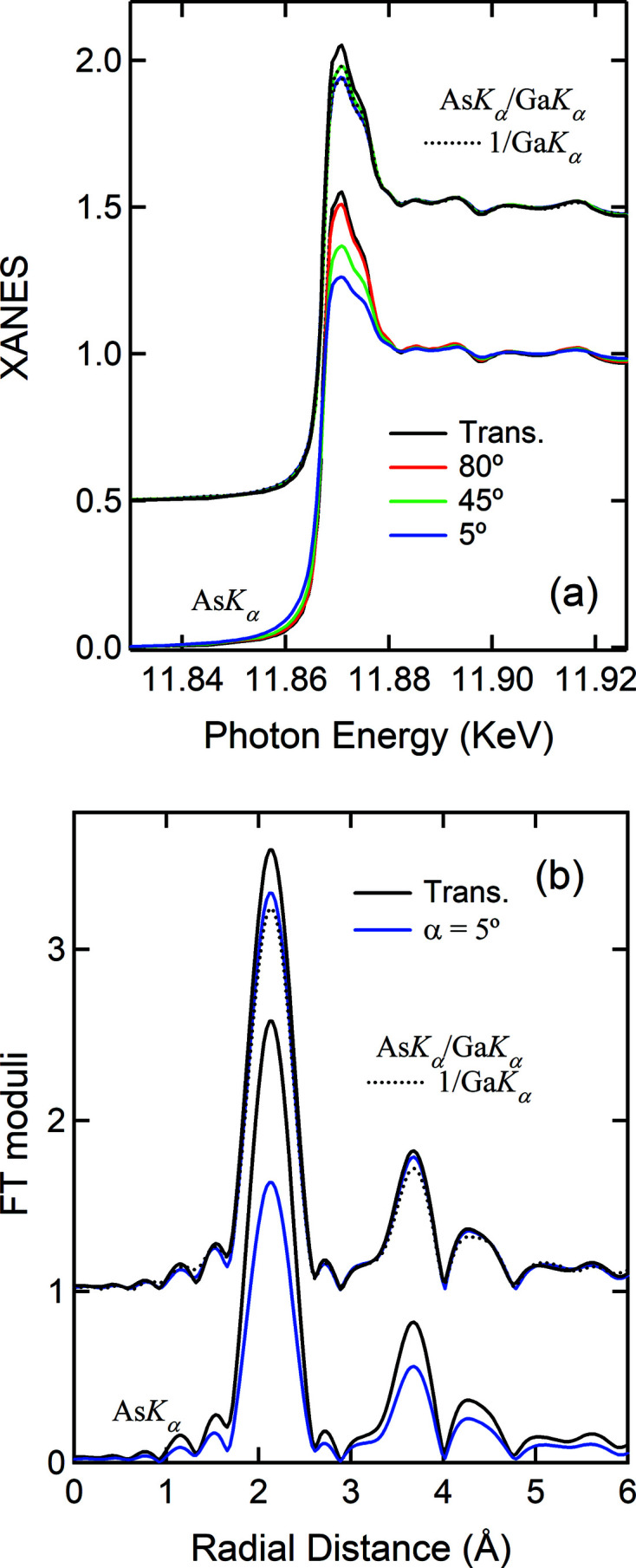
XAS spectra measured on GaAs wafer through the As *K_α_* and Ga *K_α_* channels at the As *K*-edge. All compared with the standard (black line). (*a*) Lower: XANES from As *K_α_* at α = 5°, 45°, and 80°. Upper: XANES from 1/Ga *K_α_* and As *K_α_*/Ga *K_α_, α* = 5° and 45°. (*b*) The FT moduli of the corresponding EXAFS spectra (*k* = 3–12.4 Å^−1^, Hanning window).

As was seen in Fig. 5 ▸(*b*), the SA effects show a strong angular dependence for the GaAs wafer and the best Ga *K_α_* data may be measured at low incidence angles, *e.g.* in the vicinity of α = 5°, due to the strong SA effects. The processed XAS data measured on the GaAs wafer are presented in Fig. 7 ▸ and Fig. S8. When compared with the powder [Fig. 6 ▸(*a*)], the white lines from As *K_α_* measured at α = 5° and 45° show more attenuation. Yet, in this case, the normalization method is also able to recover much of the attenuation. In contrast to the powder, both methods produce results practically indistingushable regarding the white lines. However, unlike XANES, the FT moduli [Fig. 7(*b*) ▸] show a difference of ∼5% between the As *K_α_*/Ga *K_α_* and 1/Ga *K_α_* data. Fig. S8(*a*) shows the EXAFS data measured at α = 5° and 80°. Given the better data quality, only the α = 5° data are used for the first-shell EXAFS comparison [Fig. S8(*b*)]. Careful examination [middle, Fig. S8(*b*)] shows that the As *K_α_* and 1/Ga *K_α_* data indeed disperse with *k*, albeit small, causing the difference seen in Fig. 7 ▸(*b*). This is consistent with the results presented for the powder [Fig. 6 ▸(*d*)] where a large effect is seen.

**Figure 8:**
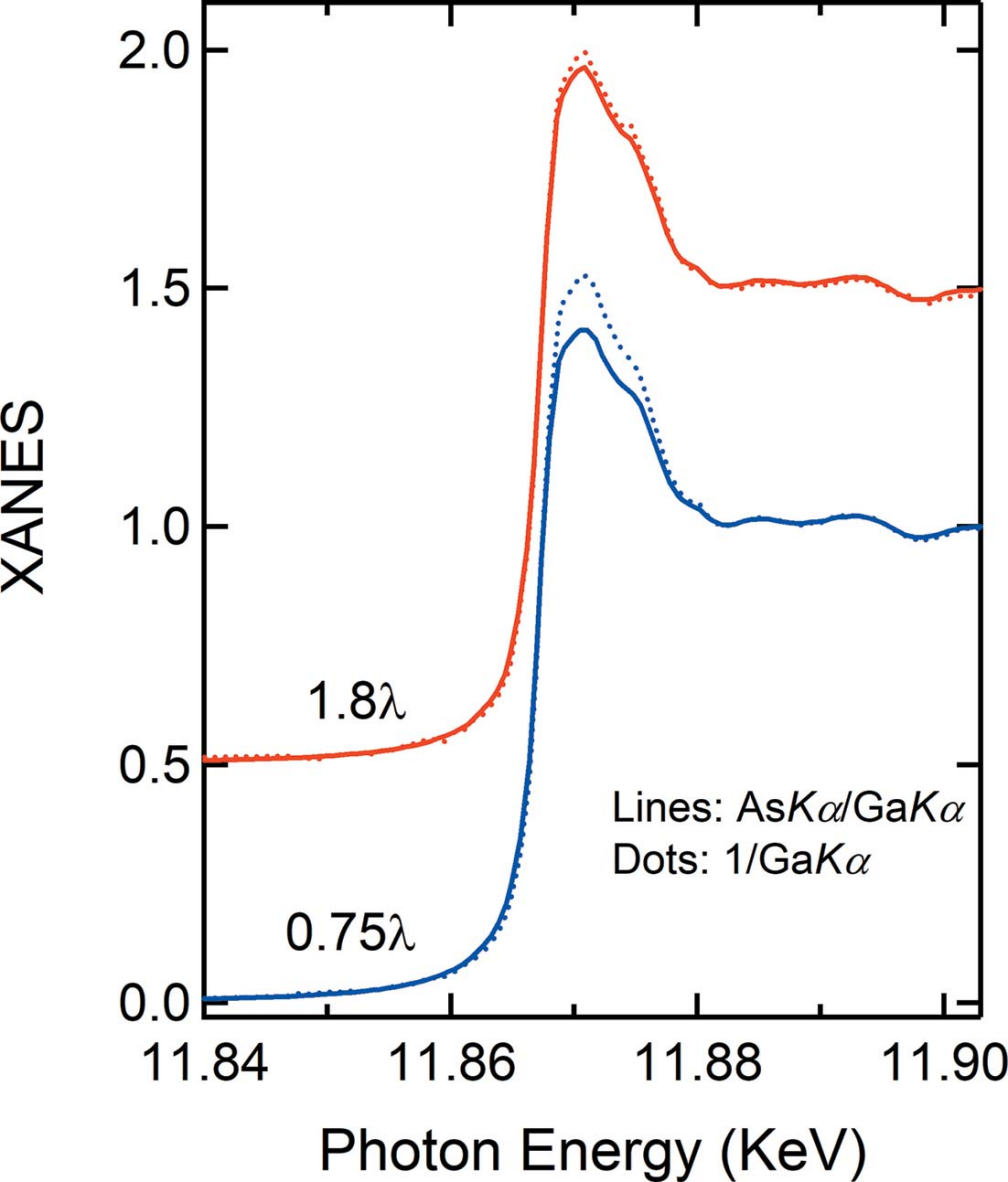
The sample thickness effects on the XANES spectra obtained using the normalization method and the IPFY method. The fluorescence data of GaAs powder were measured on the powder-on-tape samples whose optical thickness is 0.75λ (Fig. 6 ▸) and 1.8λ (Fig. S9), respectively.

The likely reason as to why both methods work almost equally well for the GaAs wafer may lie fundamentally in the fact that the half-millimetre-thick wafer is much thicker than the thin films and the powder-on-tape samples. Thus there is an optical thickness effect. The wafer is effectively infinitely thick for X-ray energies around the As *K*-edge. Therefore, the assumption of the thick limit used to reduce equation (1) to equation (2) holds up differently for the two methods in comparison. To validate this conjecture, XAS measurements were carried out on a powder-on-tape GaAs sample (Fig. S9), whose optical thickness is more than double (×2.4). As compared in Fig. 8 ▸, the 1/Ga *K_α_* data converge towards the As *K_α_*/Ga *K_α_* data. This therefore confirms that the IPFY method is susceptile to thickness effects and shall have limited applicability. On the contrary, as demonstrated in every scenario, our normalization method does not suffer from the thickness effect and can thus be broadly applied. Moreover, it is far superior in terms of data statistics and other factors. Although the noisy Ga *K_α_* signal [green dotted line in Fig. 6 ▸(*b*)] is involved in obtaining the normalized spectrum, the latter is not inferior to the As *K_α_* signal in terms of data statistics [Fig. 6 ▸(*b*)].

### 3.3. Scaling constant ϕ

We have described the principle of the normalization method and demonstrated its effectiveness in removing the SA-induced nonlinearity. Despite the underestimation of coordination numbers which is described by ϕ in equation (7), the method should allow accurate determination of bond distances and structural disorder. For a thick wafer, the underestimation varies with the measuring geometry from ϕ ≃ 1.13 at α ≃ 5° towards ϕ ≃ 1 at α = 80° [Fig. 4 ▸(*b*)]. At the 45°/45° geometry, ϕ ≃ 1.08. ϕ has a weak angular dependence for α ≤ 45°. To avoid the geometrical effect the measurement geometry remained at 45°/45° for the powder samples and fluorescence signals exiting from the front surface were collected. For the powder-on-tape GaAs samples, ϕ ≃ 1.12. This is encouraging that ϕ remains constant for a large increase (×2.4) in the optical thickness. To test our method on a broader range of samples, we also measured a powder-on-tape CuSe sample of about two optical thicknesses. The details are given in Fig. S3, where the effectiveness of the normalization method in dealing with inhomogeneous EXAFS samples and electronic noise are better demonstrated. For this sample, ϕ ≃ 1.16. Albeit of poor sample quality, the result is likely creditable, given the effectiveness of our method in dealing with imperfections. Therefore, for the spectra collected at the 45°/45° geometry, ϕ ≃ 1.08–1.16 or 1.12 ± 0.04. Given the conjugate reationship between the primary emissions from κ and ξ and given the facts that the secondary emission intensity shall increase with the increase of 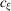 but decrease with the decrease of 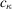 (= 1 − 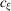 ) or 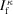 , we speculate that ϕ may not exhibit a strong stoichiometry dependence.

A common characteristic for the bulk samples studied here is that the metallic absorbers are close to each other in the periodic table, *i.e.* 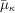 ≃ 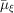 . For bulk samples with the involved elements far apart, the escaping depth effect for emitted fluoresence could come into play; it matters little for thin films like we have presented here. This will be the subject of further investigation.

When ϕ ≃ 1 as in the cases of the thin films, both XANES and EXAFS are restored (Fig. 3 ▸). For ϕ > 1, the amplitude may be recovered, if the secondary emission can be properly measured. Our first thought was to measure it on a very thin sample. In reality, however, it is much more complicated than this intuitive thought for bulk samples.^1^ As in the case of the measurements on the GaAs wafer at α = 80°, the secondary emission in fact suffers from SA effects [inset, Fig. 5 ▸(*b*)]. Since the SA effect is α-dependent, it is likely that the secondary emissions at shallow angles differ from that measured at α = 80°. Detailed in Section S10 is an exercise of using the secondary Ga *K*_α_ emission measured at α = 80° [Fig. 5 ▸(*b*)] to carry out an amplitude correction for the As *K_α_*/Ga *K*_α_ data at α = 5°. ϕ is reduced to 1.07 from 1.13. This indicates that the actual secondary Ga *K*_α_ emission at α = 5° differs both in amount and spectral shape. Therefore, the amplitudes are only partially restored here.

## 4. Conclusions

We have proposed a new route to obtain X-ray absorption spectra through fluorescence channels. We have described and demonstrated this new method analytically and experimentally. It is easy to implement experimentally and removes nonlinear spectral distortions induced by SA effects which occur in certain measuring geometries and in element-of-interest (κ) concentrated samples. The key to this method is the recognition of correlations under SA conditions among emission events following resonance X-ray core-electron excitation within κ. The intensities (*I*_f_) of fluorescence emitted from κ and from other elements ξ present in the sample are attenuated by SA effects in a conjugated fashion. Therefore, the arithmetic division of 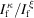 may remove SA-induced spectral distortions. However, the so-called secondary emission, usually weak, emitted from ξ under excitation of 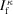 and embedded in 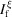 with an antiphase, induces a linear amplitude reduction. For a 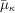 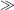 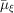 system measured under the grazing incidence geometry, this simple normalization is sufficient to restore spectral integrity with remarkable accuracy, as manifested for Ga–In–O and Zn–Sn–O thin films. It is a powerful method for studies of surface and sub-surface structures around cations which are otherwise inaccessible due to severe SA effects. For measured bulk samples with 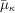 ≃ 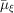 , the linear reduction due to the secondary emission amounts to ϕ = 1.12 ± 0.04%. Clearly more work is needed to see how strongly ϕ depends on stoichiometry.

This approach is entirely experimental and needs little effort to implement. It represents a technical breakthrough in tackling the SA problem through experimental means and is broadly applicable to compounds. In addition, its ability to handle sample inhomogeneity, diffraction effects, and instrument-related errors make it unique and beneficial to broad materials research communities.

## Supplementary Material

Sections S1 to S10; Figures S1 to S10; Tables S1 to S3. DOI: 10.1107/S1600577522000029/rv5154sup1.pdf

## Figures and Tables

**Table 1:** Terms in the denominators of equations (3) and (4) for α/θ = 0.1°/20°†

Energies (eV)	μ_tot_(*E*) (cm^−1^)	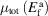 (cm^−1^)	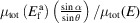
In *K*-edge + 50	126.6		
In *K_α_* (24120)		40.6	0.0016
Ga *K_α_* (9241)		460.0	0.0185

† Table S3 for GaAs.

## Footnotes

1 We were able to measure a secondary emission spectrum on a thin GaAs sample, which seems to work for recovery of the white line. However, in attempts to prepare a thin sample for CuSe powder, we failed to have a sample that is appropriate enough to have the secondary emission from Cu *K*_α_ measured. As demonstrated for GaAs wafer, the results would be erroneous in the end.

## References

[bb1] Achkar, A. J., Regier, T. Z., Wadati, H., Kim, Y., Zhang, H. & Hawthorn, D. G. (2011). *Phys. Rev. B*, **83**, 081106.

[bb2] Becker, R. S., Golovchenko, J. A. & Patel, J. R. (1983). *Phys. Rev. Lett.* **50**, 153–156.

[bb3] Booth, C. H. & Bridges, F. (2005). *Phys. Scr.* T**115**, 202–204.

[bb4] Brewe, D. L., Pease, D. M. & Budnick, J. I. (1994). *Phys. Rev. B*, **50**, 9025–9030. 10.1103/physrevb.50.9025 9974943

[bb5] Buchholz, D. B., Ma, Q., Alducin, D., Ponce, A., Jose-Yacaman, M., Khanal, R., Medvedeva, J. E. & Chang, R. P. H. (2014). *Chem. Mater.* **26**, 5401–5411. 10.1021/cm502689x PMC4311939 25678743

[bb6] Eisebitt, S., Böske, T., Rubensson, J. E. & Eberhardt, W. (1993). *Phys. Rev. B*, **47**, 14103–14109. 10.1103/physrevb.47.14103 10005751

[bb7] Ginley, D. S., Hosono, H. & Paine, D. C. (2011). *Handbook of Transparent Conductors.* New York: Springer.

[bb8] Goulon, J., Goulon-Ginet, C., Cortes, R. & Dubois, J. M. (1982). *J. Phys. Fr.* **43**, 539–548.

[bb9] Haskel, D. (1999). *FLUO: Correcting XANES for Self-Absorption in Fluorescence Measurements*, http://www.aps.anl.gov/~haskel/fluo.html.

[bb10] Moffitt, S. L., Buchholz, D. B., Chang, R. P. H., Mason, T. O., Marks, T. J., Bedzyk, M. J. & Ma, Q. (2018). *J. Phys. Chem. C*, **122**, 28151–28157.

[bb11] Moffitt, S. L., Ma, Q., Buchholz, D. B., Chang, R. P. H., Bedzyk, M. J. & Mason, T. O. (2016). *J. Phys. Conf. Ser.* **712**, 012116.

[bb12] Moffitt, S. L., Zhu, Q., Ma, Q., Falduto, A. F., Buchholz, D. B., Chang, R. P. H. R. P. H., Mason, T. O., Medvedeva, J. E., Marks, T. J. & Bedzyk, M. J. (2017). *Adv. Electron. Mater.* **3**, 1700189.

[bb13] Pfalzer, P., Urbach, J., Klemm, M., Horn, S., denBoer, M. L., Frenkel, A. I. & Kirkland, J. P. (1999). *Phys. Rev. B*, **60**, 9335–9339.

[bb14] Ravel, B. & Newville, M. (2005). *J. Synchrotron Rad.* **12**, 537–541. 10.1107/S0909049505012719 15968136

[bb15] Sayers, E. A., Stern, E. A. & Lytle, F. W. (1974). *Phys. Rev. Lett.* **27**, 1204–1207.

[bb16] Trevorah, R. M., Chantler, C. T. & Schalken, M. J. (2019). *IUCrJ*, **6**, 586–602. 10.1107/S2052252519005128 PMC6608621 31316803

[bb17] Tröger, L., Arvanitis, D., Baberschke, K., Michaelis, H., Grimm, U. & Zschech, E. (1992). *Phys. Rev. B*, **46**, 3283–3289. 10.1103/physrevb.46.3283 10004043

[bb18] Zschech, E., Tröger, L., Arvanitis, D., Michaelis, H. G. U., Grimm, U. & Baberschke, K. (1992). *Solid State Commun.* **82**, 1–5. 10.1103/physrevb.46.3283 10004043

