# Supplementary material for: A new route to obtain fluorescence X-ray absorption spectra of compounds and to remove the self-absorption induced nonlinearity in the spectra
Source: J Synchrotron Radiat. 2022 Feb 8;29(Pt 2):470–9. doi: 10.1107/S1600577522000029 (PMC8900835; doi:10.1107/S1600577522000029)
Supplement: Supplementary file 1 [file s-29-00470-sup1.pdf]

JOURNAL OF SYNCHROTRON RADIATION

Volume 29 (2022)

Supporting information for article:

A new route to obtain fluorescence X-ray absorption spectra of compounds and to remove the self-absorption induced nonlinearity in the spectra

Qing Ma, Stephanie L. Moffitt and Denis T. Keane

Supplemental materials to:

A new route to obtain fluorescence X-ray absorption spectra of compounds and to remove the self-absorption induced nonlinearity in the spectra

Qing Ma[*,1], Stephanie L. Moffitt[2], Denis T. Keane[1,2]

1. Northwestern Synchrotron Research Center at the Advanced Photon Source, Argonne, Illinois, 60439, USA

2. Materials Science and Engineering Department, Northwestern University, Evanston, Illinois 60208, USA

Content Page

S1	Film surface conditions	2
S2	Characterization of the powder-on-tape GaAs sample	2
S3	Characterization of the powder-on-tape CuSe sample	3-4
S4	The In K-edge XANES measured via the In $L_{\alpha\beta}$ channel	5
S5	Fourier transforms of the In K-edge EXAFS	5
S6	The results for an amorphous Zn-Sn-O film	6
S7	Effect of the γ factors for Ga-In-O and GaAs: $\gamma = \frac{\mu_{tot}(E)+\mu_{tot}(E_f^{Ga})(\frac{\sin\alpha}{\sin\theta})}{\mu_{tot}(E)+\mu_{tot}(E_f^{In})(\frac{\sin\alpha}{\sin\theta})}$	7-8
S8	More analyses of the EXAFS spectra measured on a GaAs wafer	8
S9	The XAS data of two GaAs samples with different thicknesses	9
S10.	The EXAFS amplitude correction by removing the secondary emission	10

[*] The author to whom the correspondence should be addressed to: q-ma@northwestern.edu

S1. Film surface conditions

Figure S1 shows the surface conditions of the amorphous Ga (10%)-In-O and Zn (30%)-In-O films, respectively, shown by the X-ray reflectivity (XRR) and angular dependence of X-ray fluorescence (XRF) emissions. The differences in the curve shapes are attributed to the surface roughness, meaning that the surface of the Ga-In-O film is rougher than that of the Zn-Sn-O film. A preliminary atomic force microscope (AFM) image measured on the Ga-In-film in a surface area of 4×4 μm^2 shows a mean roughness of 3.4 nm, which is comparable to the probing depth when the X-ray incidence angle is smaller than the critical angle. In addition, the film surface shows micron-sized unevenness, a condition that is primarily responsible for the gently sloping XRR/XRF curves shown on the left in Fig. S1. Consequently, the In *K*-edge XANES measured at a ultra-small incidence angle α (~0.06°) via the In K_α channel suffers severe SA-effect (see Fig. 3a) in comparison to that measured at a similar angle on the Zn-Sn-O film (Fig. S5).

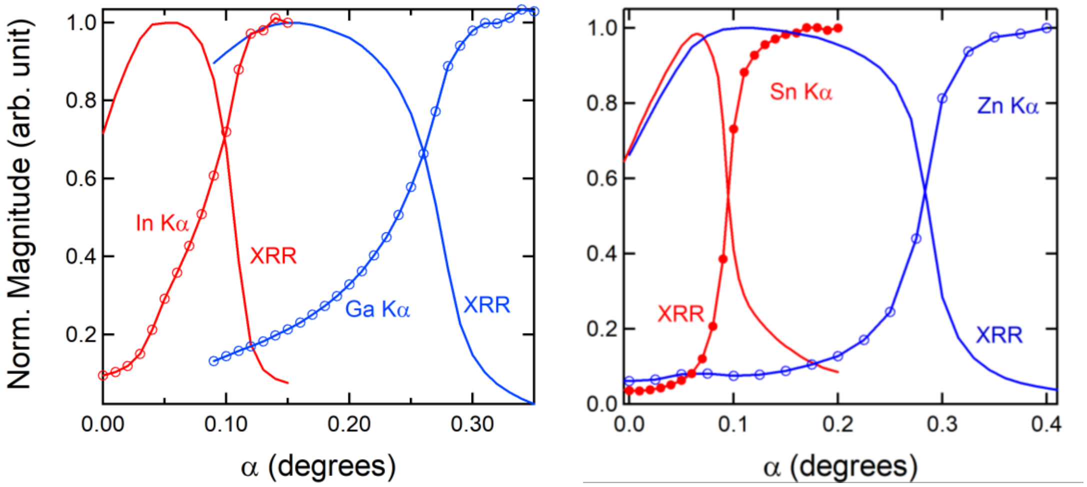

Figure S1. XRRs and XRFs of the Ga-In-O film (left) and Zn-Sn-O film (right). The data are measured at 50 eV above the Ga (Zn) and In (Sn) *K* edges, respectively.

S2. Characterization of the powder-on-tape GaAs sample

A fine powder obtained from the GaAs wafer is used as a standard and is prepared on Scotch tape which is then folded a few times for measurements in transmission mode. The sample uniformity is crucial for the XAS measurements in transmission mode. Therefore, the prepared sample is checked with a small X-ray beam (0.2×1.2 mm^2) by scanning the sample while monitoring the transmitted beam intensity. As

shown in Fig. S2, the variation in the intensity is ≤ 2% (SD/AM) within the beam spot. In addition, the beam-size-dependent measurements were carried out to illustrate the effect of the sampling size. The results are shown in Fig. S2-a, b, c, and d. Once the beam size is > 4 mm the data quality is no longer dependent on sampling size. The sample homogeneity issue will be a concern again in S3 for the CuSe powder.

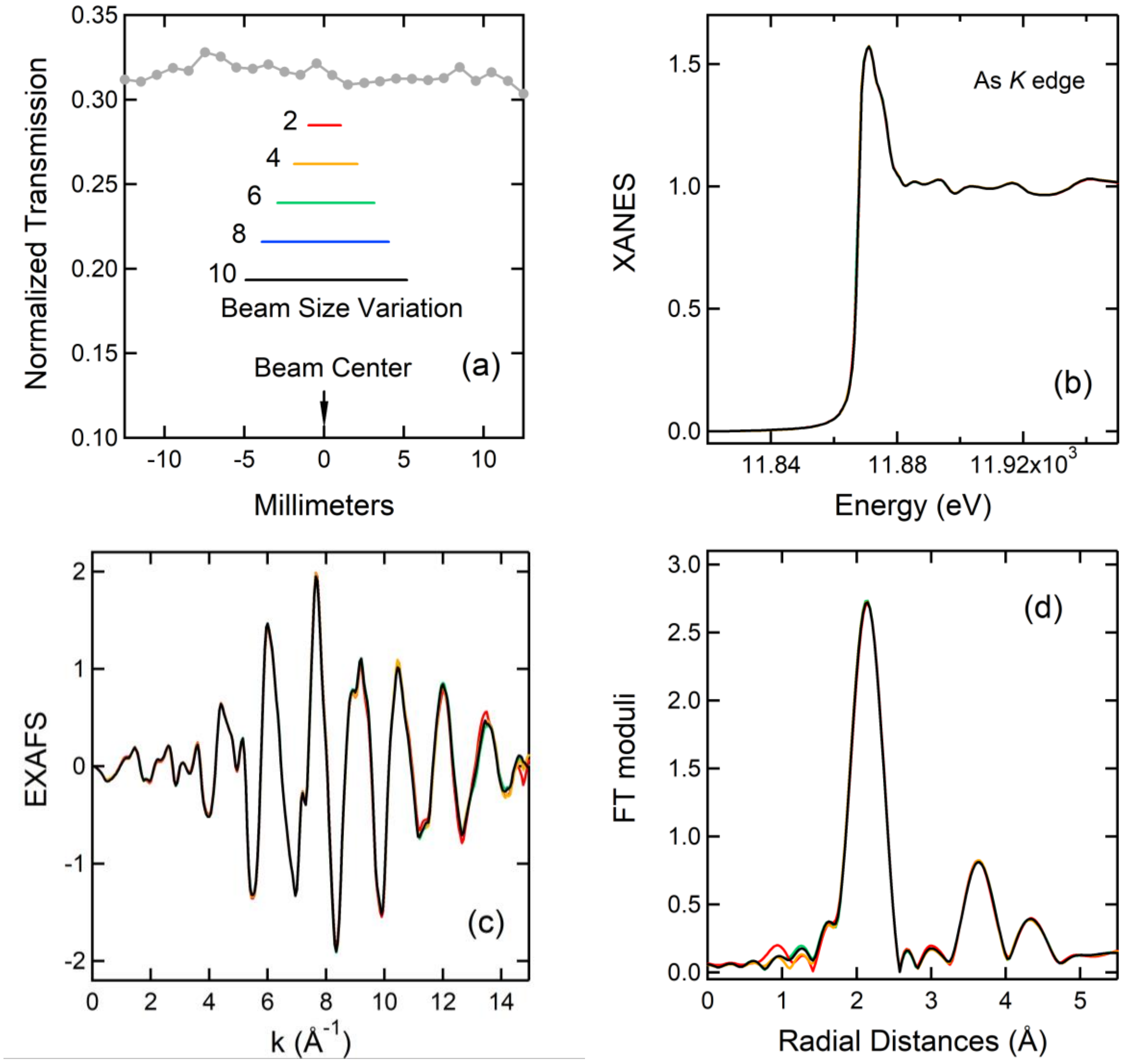

Figure S2. The GaAs powder sample uniformity check. (a) The transmitted intensities measured as the sample moves across the $0.2\times1.2\ mm^2$ beam. (b) the measured XANES using the beam sizes given in (a). (c) the extracted 5 EXAFS spectra. (d) the FT moduli using the data from 3 to 13.2 $Å^{-1}$.

S3. Characterization of the powder-on-tape CuSe sample

For some physical reason it was hard to prepare a quality CuSe powder sample. However, a powder-on-tape sample is used to illustrate the common problems with inhomogeneous samples when measured in transmission mode and to demonstrate the power of the normalization method introduced in this work. As shown in Fig. S3-a, the data show strong irregularities when measured in transmission mode using a small

beam ($1.0\times1.5\ mm^2$) on different spots. The average compares favorably to the data using a large beam of $1.0\times8\ mm^2$ which may not be free of some distortion. Fig. S3-b shows the EXAFS spectra from the normalization method, i.e., SeK_α/CuK_α. Their average is compared to the transmission data which is scaled by ×0.86, suggesting an EXAFS amplitude reduction by a constant of 1.16 (or 1/0.86). Their FTs are compared in Fig. S3-c. Figure S3-d is used to demonstrate the power of the normalization method in dealing with electronic anomalies, large or small. Irregularities appear both in the Se K_α and Cu K_α data measured simultaneously. However, it is canceled out by SeK_α/CuK_α (at ~9.35 $Å^{-1}$).

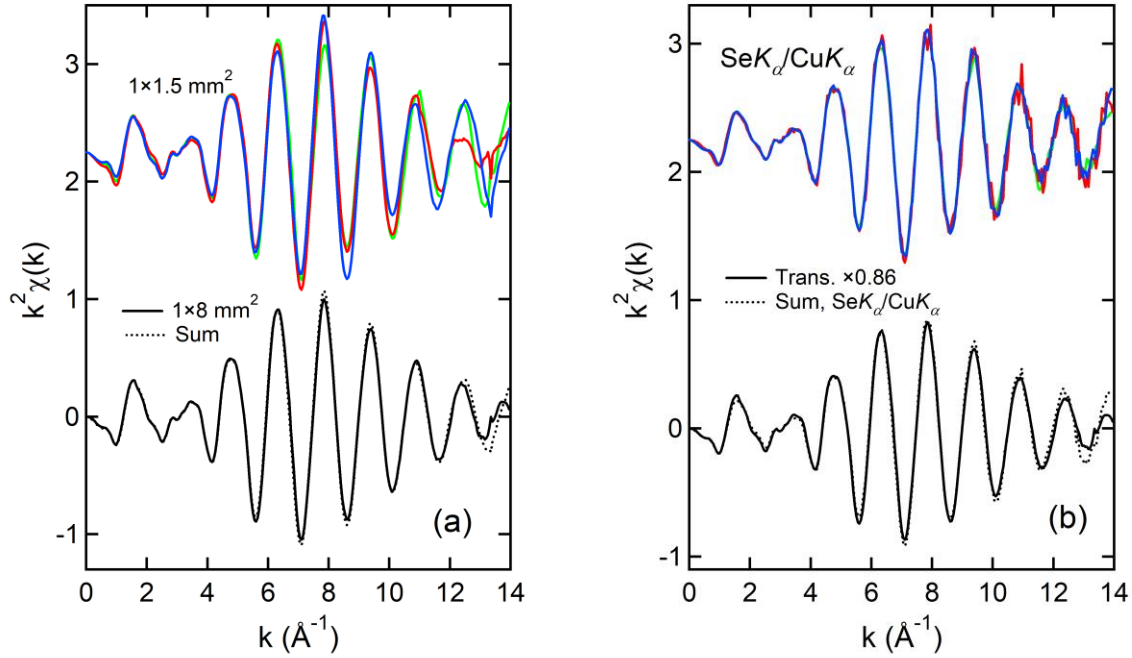

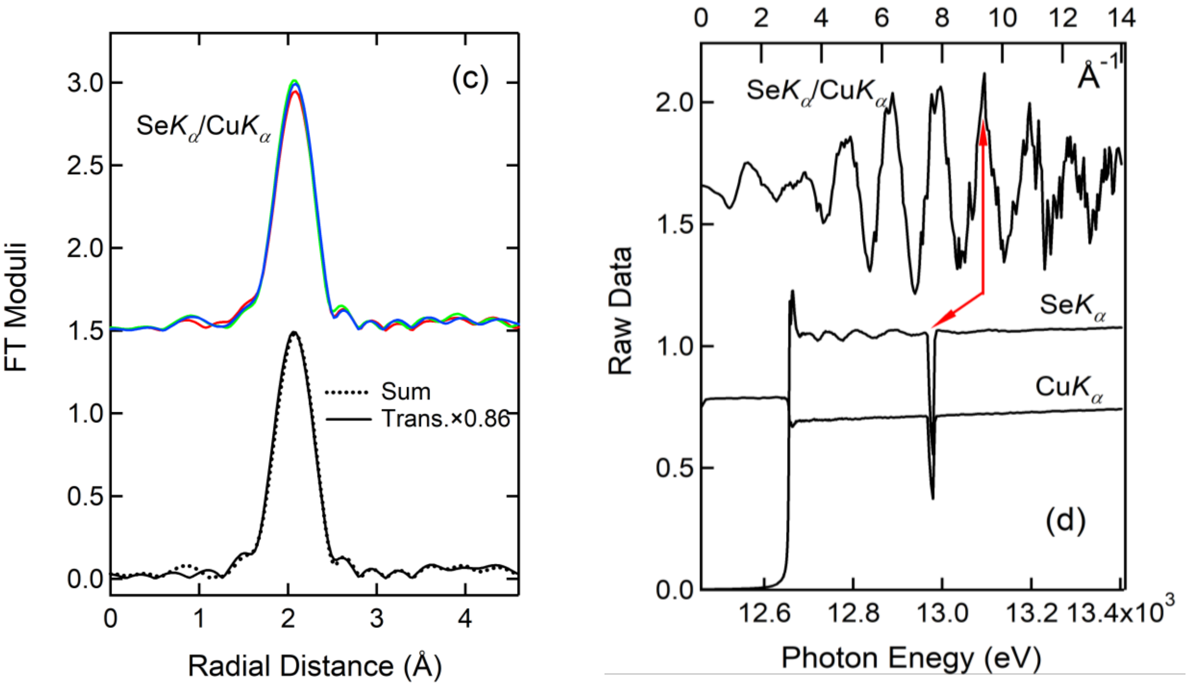

Figure S3. Powder-on-tape CuSe. (a) EXAFS spectra (upper) collected on three spots using a small beam. The average (lower) compared to the standard measured using a large beam. (b) the EXAFS spectra from SeK_α/CuK_α (upper). The average is compared to the scaled standard. (c) the FT moduli from (a) and (b). (d) One example to show the normalization in removing electronic anomalies.

S4. Fourier transforms of the In *K*-edge EXAFS

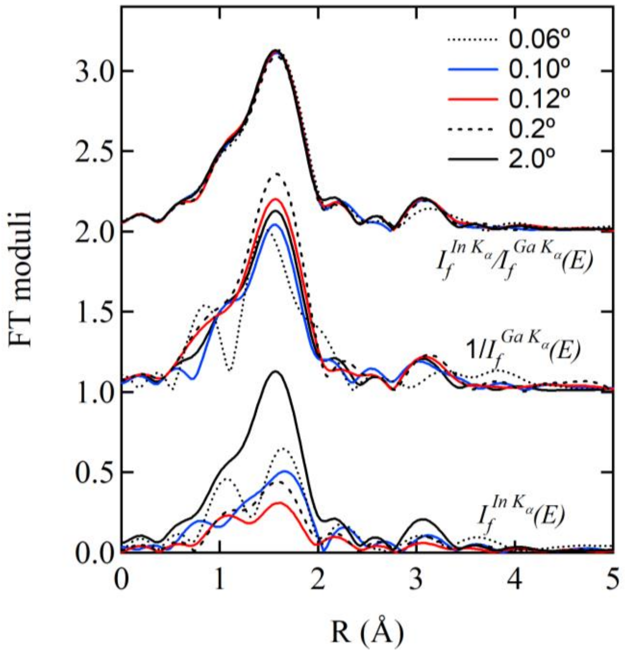

Figure S4. Comparison of the Fourier transforms of the EXAFS spectra (k = 2.2 to 10.0 Å^{-1}) obtained in three ways and presented in Fig. 2(b) in the main text. At the bottom of the figure is the conventional $I_f^{InK_\alpha}(E)$, in the middle the IPFY method $1/I_f^{GaK_\alpha}(E)$, and at the top the new normalization method $I_f^{InK_\alpha}/I_f^{GaK_\alpha}$. Compared to the In K_α emission data (α = 2°), there are large differences among the spectra (α = 0.1° to 0.2°) obtained by two other methods.

S5. The In *K*-edge XANES measured via the In $L_{\alpha\beta}$ channel.

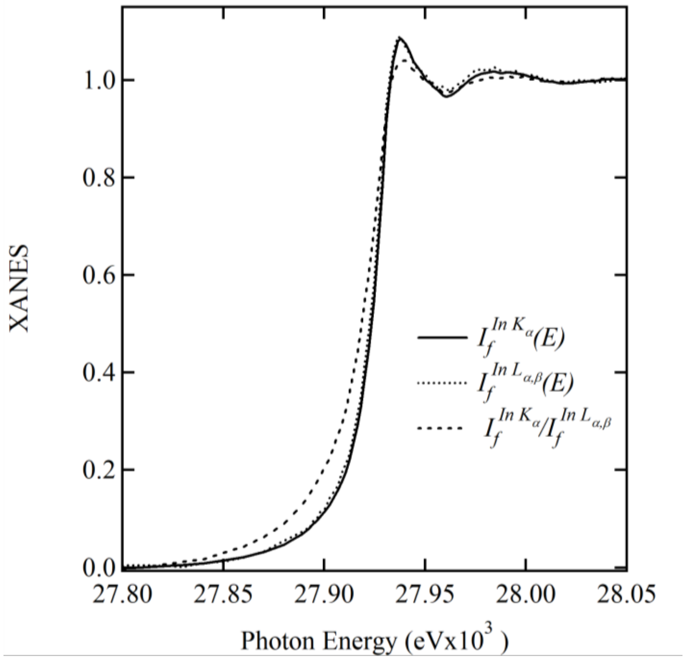

Figure S5. Comparison of the In *K*-edge XANES data measured simultaneously *via* the In K_α and $L_{\alpha\beta}$ channels with α = 0.12°. The data are normalized by the edge step heights. Also shown is the ratio of these two data sets. Contrary to the suggestions by Achkar *et al*. [1], inversion of the In $L_{\alpha\beta}$ emission (the IPFY method) will produce unphysical results.

S6. The results for an amorphous Zn-Sn-O film

Both the IPFY method and the normalization method introduced here are also applied to the Zn-Sn-O film with ~30 at. % Zn. As indicated by the XRR and XRF data presented in Fig. S1, the surface of the Zn-Sn-O film is likely smoother. The $\mu_{tot}(E_f^{\alpha})(\frac{\sin\alpha}{\sin\theta})/\mu_{tot}(E)$ term (see Table 1 in the main text) is 0.002 and 0.025, respectively, for Sn K_α and Zn K_α. Therefore, Eq. 6 can be well applied to the Zn(30%)-Sn-O film. It is estimated from the data measured at α = 2° that $I_s^{ZnK_\alpha}(E)$ is ~6.5 % of $I_f^{ZnK_\alpha}(E)$. The quantum yield of $I_s^{ZnK_\alpha}(E)$ due to $I_f^{SnK_\alpha}(E)$ is ~1%, which will be lower by about one order of magnitude if the quantum efficiency of the Si-drift detectors is considered. The SA effects are severe for 0.07° < α < 2°. As for the Ga-In-O film, the new normalization method clearly produces physically sound results.

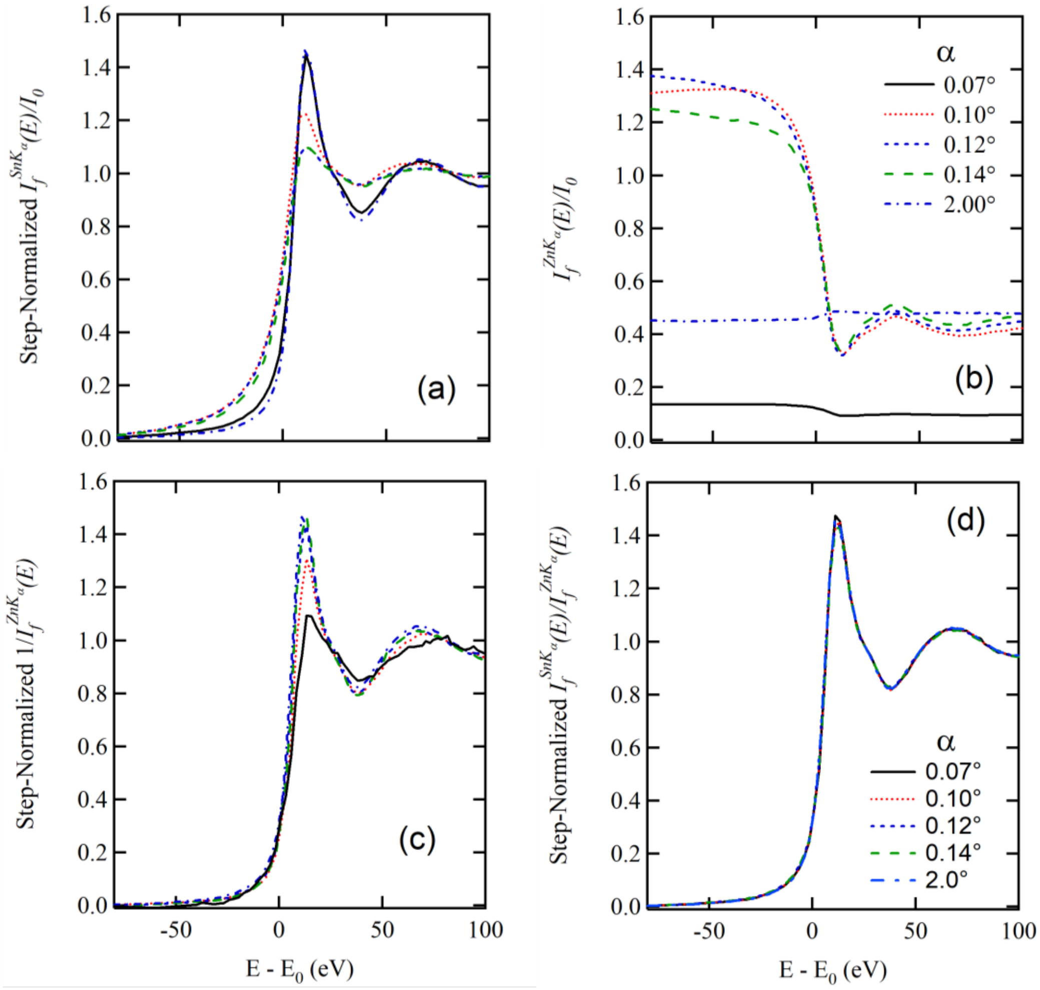

Figure S6. The Sn *K*-edge XAS measurements of the Zn-Sn-O film vs α. (a) The $I_f^{SnK_\alpha}(E)$ spectra normalized by their step heights. (b) The $I_f^{ZnK_\alpha}(E)$ (raw data). (c) $1/I_f^{ZnK_\alpha}$ and then normalized to their step heights. (d) the $I_f^{SnK_\alpha}/I_f^{ZnK_\alpha}$. The XRR and XRF data are shown in **Fig. S1**.

S7. Effect of the γ factors for Ga-In-O and GaAs: $\gamma = \frac{\mu_{tot}(E)+\mu_{tot}(E_f^{\xi})(\frac{\sin\alpha}{\sin\theta})}{\mu_{tot}(E)+\mu_{tot}(E_f^{\kappa})(\frac{\sin\alpha}{\sin\theta})}$.

The In and As *K*-edge spectra, μ_{exp}, free of SA effects are inserted (scaled) into tabulated absorption coefficients for the γ calculation: $\mu_{exp} \times \frac{\Delta\mu_{theory}}{\Delta\mu_{exp}} + (\mu_{theory}^{E1} - \mu_{exp}^{E1}\frac{\Delta\mu_{theory}}{\Delta\mu_{exp}})$.

Table S1. Scaling the measured data to tabulated values for calculation of γ (Ga-In-O)

κ = In and ξ = Ga	E1 = 27690 eV	E2 = 28106 eV
μ_{Ga} / μ_{In} / μ_O	16 / 8.5 / 0.433	15.363 / 46.616 / 0.442

$Ga_{0.2}In_{1.8}O_3$, $\mu\times\rho$, $\rho = 7.1$ g/cm^3	28.11	125.32
$\mu_{In}\times\rho$, ρ=7.31 g/cm^3	62.13	340.76
μ_{exp}	In K-edge XAS measured at α = 5º, $\Delta\mu_{exp}$= 2.2088-0.0602	
$\Delta\mu_{theory}$	125.32-28.11	

Table S2. Scaling the measured data to tabulated values for calculation of γ (GaAs)

κ = As and ξ = Ga	E1 = 11809 eV	E2 = 11908 eV
μ_{Ga} / μ_{As}	158.858 / 26.13	155.431 / 177.714
GaAs, $\mu\times\rho$, $\rho = 5.3$ g/cm^3	490.25	882.84
$\mu_{As}\times\rho$, $\rho = 5.72$ g/cm^3	149.5	1016.5
μ_{exp}	Fine GaAs powder, $\Delta\mu_{exp}$= 0.98	
$\Delta\mu_{theory}$	882.84 - 490.25	

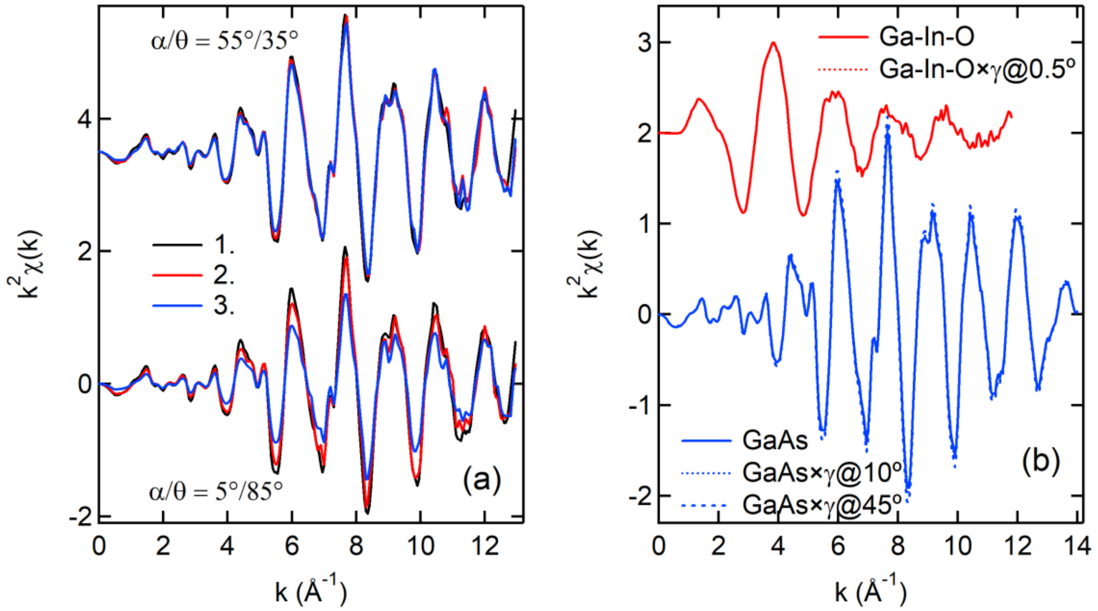

Figure S7. (a) The EXAFS spectra measured at α = 5° and α = 55° on a GaAs wafer and presented to support, as examples, the results in Fig. 4b of the main text. 1. Powder, 2. $I_f^{AsK\alpha}(E)/I_f^{GaK\alpha}(E)$, 3. $I_f^{AsK\alpha}(E)$. (b) The In *K*-edge EXAFS of the Ga-In-O film measured at α = 5° and the As K-edge EXAFS measured on GaAs powder, respectively, times the γ factors at α = 0.5° for Ga-In-O and α = 10° and 45°. γ at α = 0.5° decreased the EXAFS amplitude by ~2 % for Ga-In-O, while it increased the amplitude by ~2.0 % at α = 10° and by ~8.0 % at α = 45° for GaAs. These changes are linearly scalable.

Table S3. The terms in the denominators in Eq. 3 and 4 for α/θ =0.1º/20º for GaAs

Energies (eV)	$\mu_{tot}(E)$ (cm^{-1})	$\mu_{tot}(E_f^{a})$ (cm^{-1})	$\mu_{tot}(E_f^{a})(\frac{\sin\alpha}{\sin\theta})/\mu_{tot}(E)$
As *K* edge + 50	880.95		
As K_α (10530)		658.7	0.0038
Ga K_α (9241)		247.68	0.0014

S8. More analyses of the EXAFS spectra measured on a GaAs wafer.

Figure S8 shows the angular dependence of the EXAFS spectra measured on a GaAs wafer. The XANES and the FT moduli are presented in Fig. 7 in the main text. It will be beneficial to compare these results with the ones presented in Fig. 6 for the GaAs powder. Albeit weak, the conclusions are the same as for the powder.

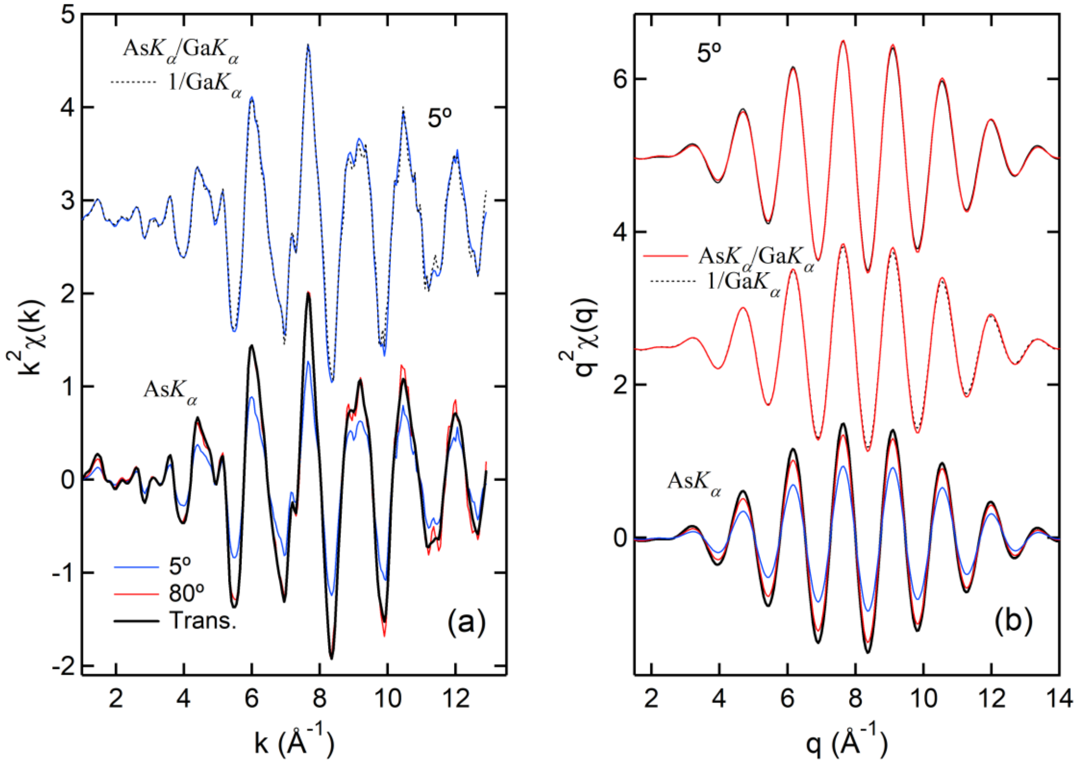

Figure S8. (a) Lower: EXAFS from AsK_α at 5° and 80°. Upper: EXAFS from 1/GaK_α and AsK_α/GaK_α α = 5°. (b) The first shell EXAFS at 5° obtained by the back FTs (R = 1.6-2.6 Å). Lower: AsK_α (blue) and AsK_α/GaK_α (red). Middle: The AsK_α and 1/GaK_α scaled in the low k region. Upper: AsK_α/GaK_α scaled to the standard by ×1.14±0.02.

S9. The XAS data of two GaAs samples with different thicknesses

Figure S9 is used to show that under an identical experimental geometry, the results obtained by the normalization method do not suffer from the thickness effect. The data are measured on two powder-on-tape GaAs samples of different optical thicknesses. This is in sharp contrast to the ones from the IPFY method (Fig. 8 in the main text).

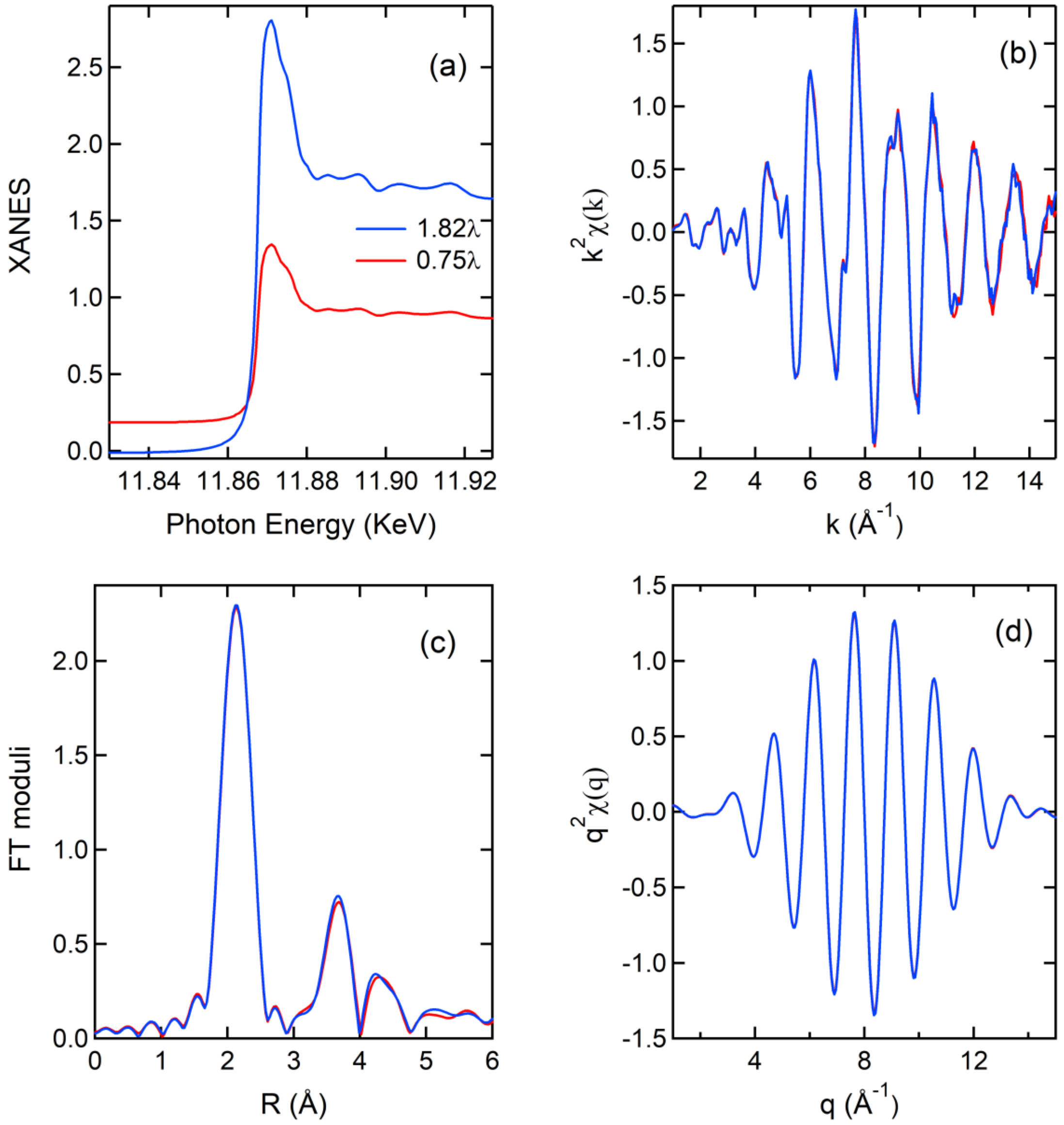

Figure S9. Comparison of the 0.75λ and 1.82 λ powder-on-tape samples. (a) XANES; (b) EXAFS from the AsK_α/GaK_α data; (c) FTs of the AsK_α/GaK_α data; (d) The first shell EXAFS's of the AsK_α/GaK_α spectra. Both results can be scaled to the standard using the same scaling constant ϕ = 1.12.

S10. The EXAFS amplitude correction by removing the secondary emission

This exercise is to show that it is not sufficient to correct the amplitude reduction in the $AsKa/GaKa$ data measured at α = 5° on the GaAs wafer using the secondary Ga K_a emission measured at α = 80° under no SA conditions (the insert, Fig. 5b). The step ratio calculated this way, $\frac{\Delta GaKa(80^{\circ})}{\Delta AsKa(80^{\circ})}$, gives the secondary emission per As K_a count, assuming that the secondary emission amount is proportional to the As K_α intensity. The secondary Ga K_α emission intensity at α = 5° is thus calculated by $\Delta GaK\alpha(5^{\circ}) = \left[\left(\frac{\Delta GaK\alpha(80^{\circ})}{\Delta AsK\alpha(80^{\circ})}\right) \times \Delta AsK\alpha(5^{\circ})\right] \times [GaK\alpha(80^{\circ}) - preedge]$. The amplitude corrected AsK_α/GaK_α data at α = 5° is AsK_α(5°)/[GaK_α(5°)-ΔGaK_α(5°)]. Comparing this data to the standard yields ϕ = 1.07.

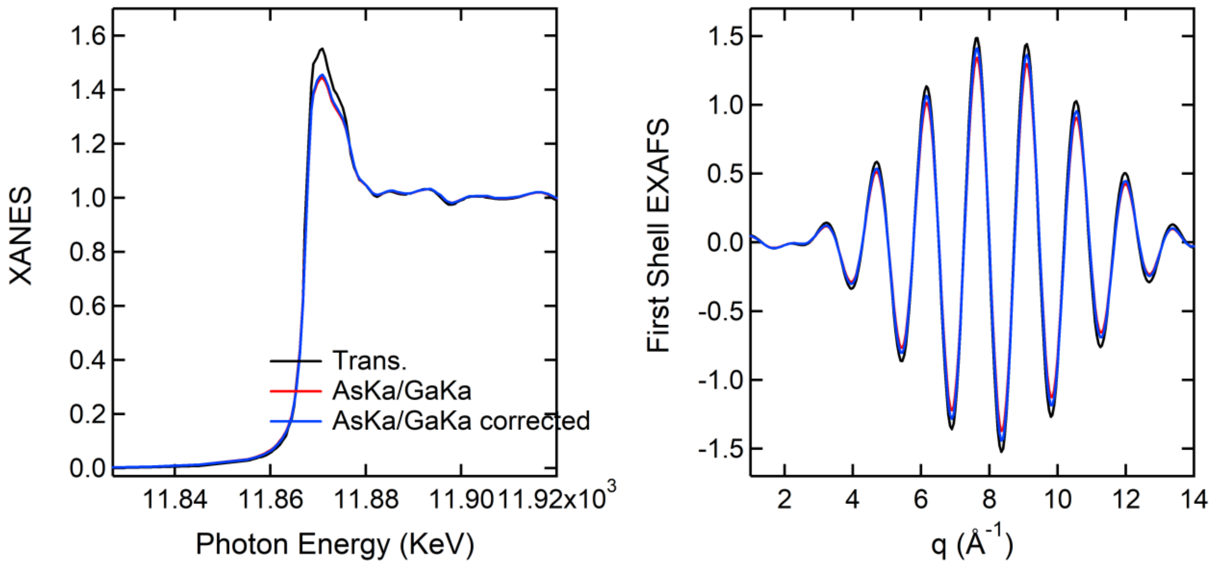

Figure S10. The AsK_α/GaK_α data measured at α = 5° are corrected by the data measured at α = 80°.
